# Supplementary figures and images for: Alix is required for activity-dependent bulk endocytosis at brain synapses
Source: PLoS Biol. 2022 Jun 3;20(6):e3001659. doi: 10.1371/journal.pbio.3001659 (PMC9200306; doi:10.1371/journal.pbio.3001659)

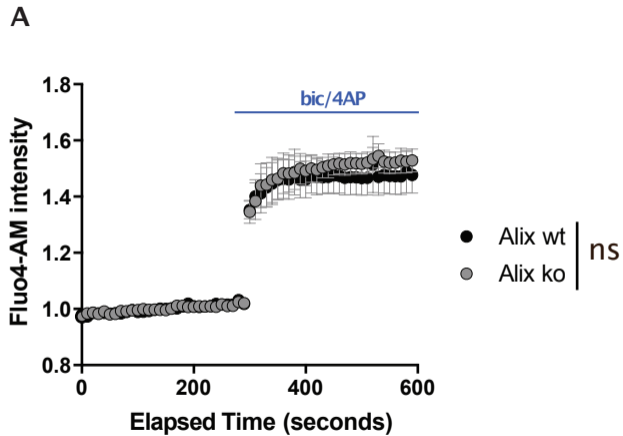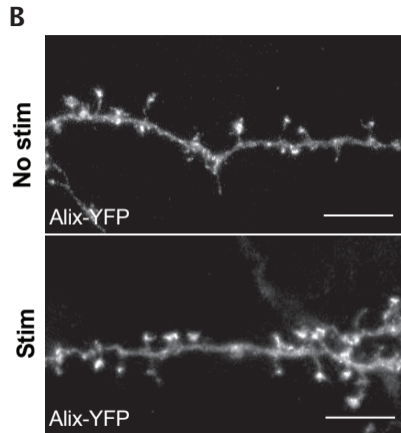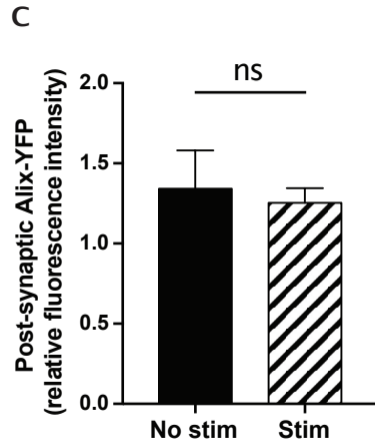

Supplement: S1 Fig — (A) No difference in calcium rise in Alix wt and ko hippocampal neurons upon Bic/4AP stimulation. Fluo4-AM intensity corresponds to the change in fluorescence, normalized to initial fluorescence (N = 4 experiments, p = 0.9999, 2-way ANOVA). (B, C) Alix-YFP does not accumulate in dendritic spines upon Bic/4AP stimulation of 15 DIV hippocampal neurons. Scale bar: 5 μm. Postsynaptic Alix-YFP corresponds to the ratio between YFP-fluorescence at PSD95 labeled ROI and at neighboring dendritic parts. Scale bar: 5 μm. Average +/‒ SEM, N, statistical analysis: (A) N = 4 experiments, p = 0.9999, 2-way ANOVA. (C) 1.34 +/‒ 0.24; 1.25 +/‒ 0.09 for no stim and stim, respectively. N = 33 and N = 25 neurons for both conditions, from 5 experiments, p = 0.3821, Mann–Whitney test. The data underlying all the graphs shown in the figure can be found in S1 Data. Alix, ALG-2-interacting protein X; DIV, day in vitro; ko, knockout; wt, wild type. (PDF) [file pbio.3001659.s001.pdf]

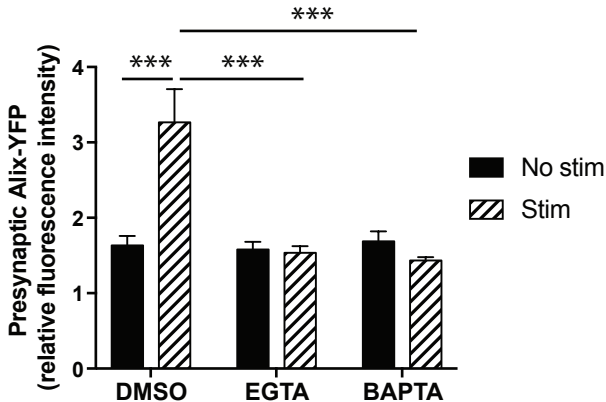

Supplement: S2 Fig — Presynaptic Alix-YFP corresponds to the ratio of fluorescence between presynaptic and non-synaptic axonal ROI. Average +/‒ SEM, N, statistical analysis: 1.63 +/‒ 0.12; 3.27 +/‒ 0.44; 1.58 +/‒ 0.10; 1.54 +/‒ 0.009; 1.69 +/‒ 0.13; 1.43 +/‒ 0.04 for DMSO no stim, DMSO stim, EGTA no stim, EGTA stim, BAPTA no stim, BAPTA stim respectively. N = 12, 9, and 9 neurons for DMSO, EGTA, and BAPTA, respectively. p = 0.0001, 1-way ANOVA. The data underlying all the graphs shown in the figure can be found in S1 Data. Alix, ALG-2-interacting protein X; ROI, Regions of interest. (PDF) [file pbio.3001659.s002.pdf]

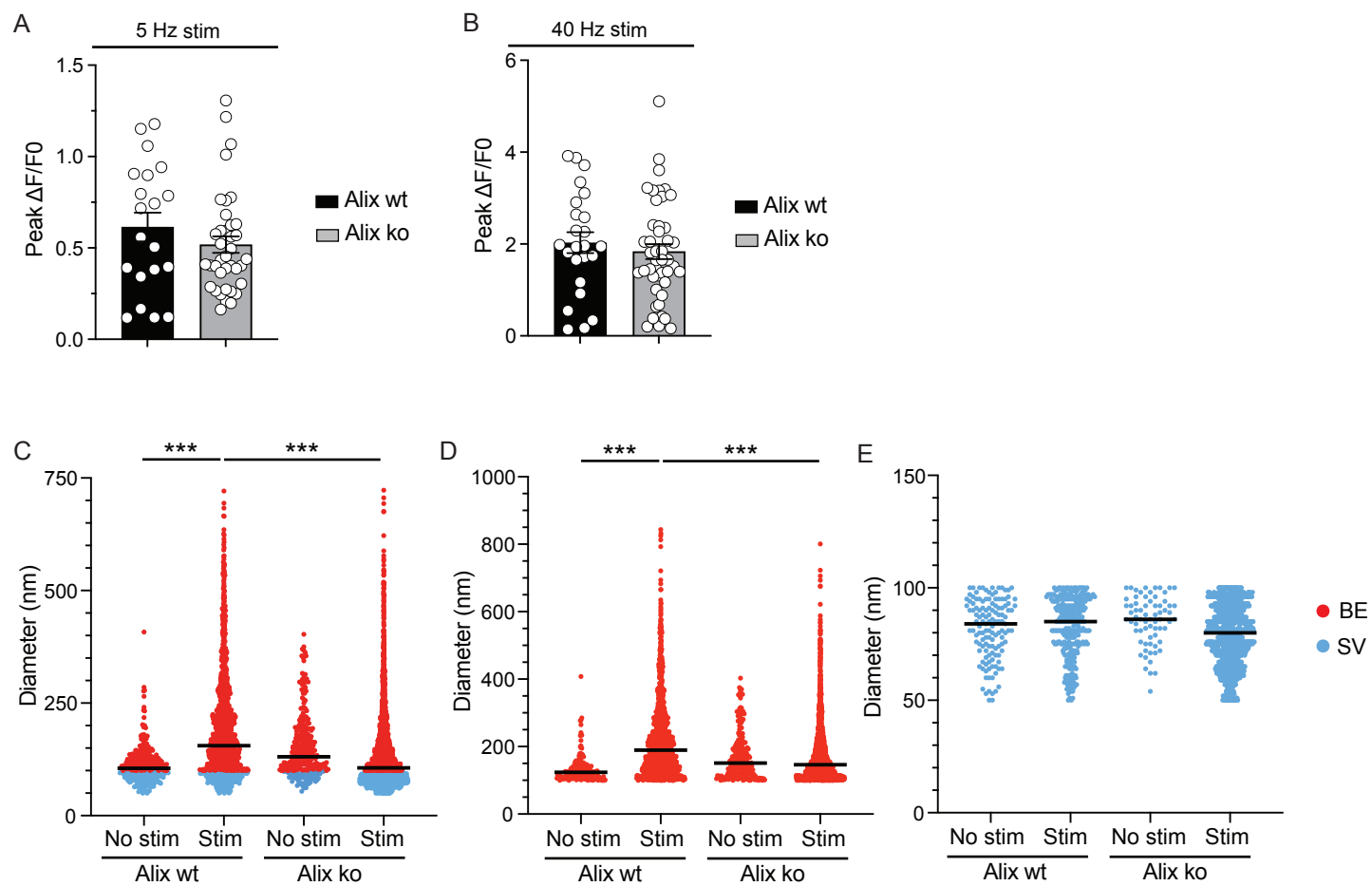

Supplement: S3 Fig — (A, B) Quantification of the syp-pH peak of fluorescence (PeakDF) during stimulation at 5 Hz (A) and 40 Hz (B) normalized to the F0 showing no significant difference in syp-pH exocytosis between Alix wt and Alix ko both at 5 Hz and 40 Hz. (C–E) Full dot plot representation of SV (blue dots C, E) and bulk endosome (red dots, C, D) diameters in Alix wt and Alix ko neurons in basal or stimulated condition showing that bulk endosomes of Alix ko synapses are smaller than in Alix wt neurons, related to Fig 3K. Average +/‒ SEM, N, statistical analysis: (A) 0.6141 +/‒ 0.078, 0.5184 +/‒ 0.044 for Alix wt and Alix ko, respectively. N = 20 and 38 for Alix wt and Alix ko, respectively, p = 0.4028, Mann–Whitney test. (B) 2.030 +/‒ 0.2281, 1.84 +/‒ 0.162 for Alix wt and Alix ko, respectively. N = 24 and 45 for Alix wt and Alix ko, respectively, p = 0.4854, Unpaired t test. (C) 112.2 +/‒ 2.37 nm; 191.7 +/‒ 3.39 nm; 152.4 +/‒ 4.1 nm; 135.2 +/‒ 1.79 nm for Alix wt no stim, Alix wt stim, Alix ko no stim, Alix ko stim, respectively. N = 304, 1,326, 285, 2,415 vesicle for Alix wt no stim, Alix wt stim, Alix ko no stim, Alix ko stim, respectively, from 3 independent experiments. p < 0.0001 in all conditions tested, Kruskal–Wallis test. (D) 133.8 +/‒ 2.99 nm; 223.3 +/‒ 3.82 nm; 171.5 +/‒ 4.6 nm; 179.9 +/‒ 2.60 nm for Alix wt no stim, Alix wt stim, Alix ko no stim, Alix ko stim, respectively. N = 181, 1,031, 223, 1,354 bulk endosomes for Alix wt no stim, Alix wt stim, Alix ko no stim, Alix ko stim, respectively, from 3 independent experiments. p < 0.0001 in all conditions tested, Kruskal–Wallis test. (E) 81.7 +/‒ 1.61 nm; 82.2 +/‒ 0.76 nm; 85.4 +/‒ 1.3 nm; 78.8 +/‒ 0.39 nm for Alix wt no stim, Alix wt stim, Alix ko no stim, Alix ko stim, respectively. N = 131, 309, 69, 1,090 SVs for Alix wt no stim, Alix wt stim, Alix ko no stim, Alix ko stim, respectively, from 3 independent experiments. The data underlying all the graphs shown in the figure can be found in S1 Data. Alix, ALG-2-interacti [file pbio.3001659.s003.pdf]

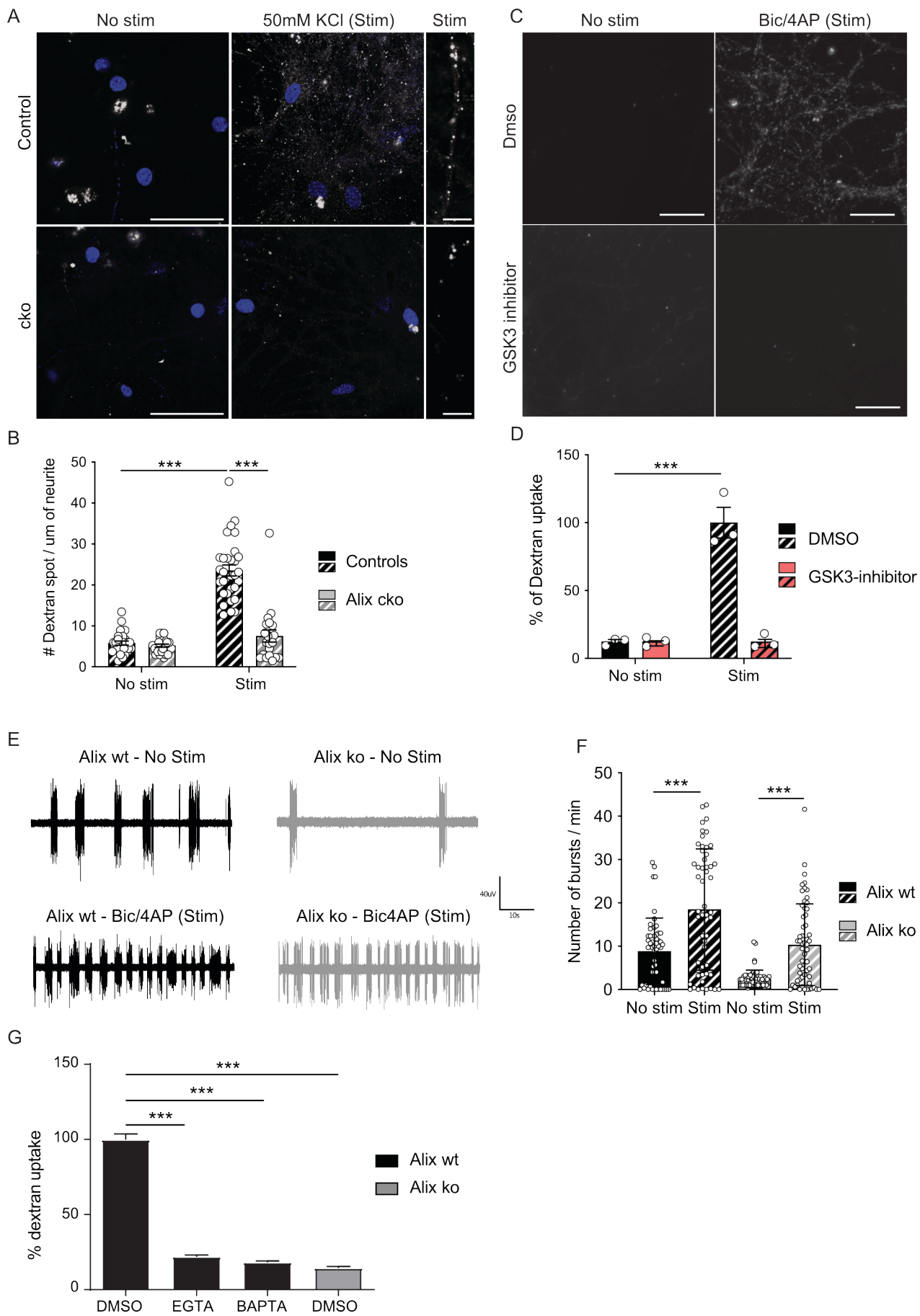

Supplement: S4 Fig — (A) Confocal images of control and Alix cko hippocampal neurons unstimulated (left) and stimulated (right + inset) in the presence of 10 kDa dextran (red). Scale bars: 50 μm and 10 μm (inset). (B) Quantification of the dextran uptake showing a significant reduction of uptake in cko neurons upon stimulation with 50 mM KCl for 90 s. (C, D) Dextran uptake is abolished in Alix wt neurons treated with an inhibitor of bulk endocytosis (GSK3-inhibitor). Confocal images of Alix wt hippocampal neurons stimulated in the presence of 10 kDa dextran with or without a GSK3 inhibitor. Scale bar: 50 μm. The % dextran uptake corresponds to the number of dextran spots per ROI expressed as percentages of the positive control. (E, F) Multiple electrode array activity recordings of 15 DIV hippocampal neuron cultures showing the effect of Bic/4AP incubation for 10 min. Representative traces of wt (black) and ko (gray) cultures are shown on panel E. (G) Dextran uptake is abolished in wt neurons by calcium chelators EGTA and BAPTA. The % dextran uptake corresponds to the number of dextran spots per ROI expressed as percentages of the positive control for each experiment. Average +/‒ SEM, N, statistical analysis: (B) 5.80 +/‒ 0.50; 5.19 +/‒ 0.39; 23.54 +/‒ 1.361; 7.529 +/‒ 1.476 for controls no stim, cko no stim, controls stim, cko stim, respectively. N = 27, 23, 30, 21 controls no stim, cko no stim, controls stim, cko stim, respectively, from 4 independent experiments, p < 0.0001, 1-way ANOVA. (C) 12.41 +/‒ 1.51; 100 +/‒ 11.29; 12.46 +/‒ 1.90; 12.40 +/‒ 2.99 for DMSO no stim, DMSO stim, GSK3-inhibitor no stim, GSK3-inhibitor stim, respectively. N = 3 experiments, p = 0.0001, 1-way ANOVA. (F) average +/‒ SEM are as follow: 8.81 +/‒ 1.05; 18.48 +/‒ 1.92; 2.41 +/‒ 0.27; 10.33 +/‒ 1.23 for Alix wt no stim, Alix wt stim, Alix ko no stim, Alix ko stim, respectively. N = 53 and 58 field of view for Alix wt and Alix ko, respectively, p < 0.0001, 1-way ANOVA. (F) 100 +/‒ 0; 21.89 +/‒ 4.19; 18.34 +/ [file pbio.3001659.s004.pdf]

A

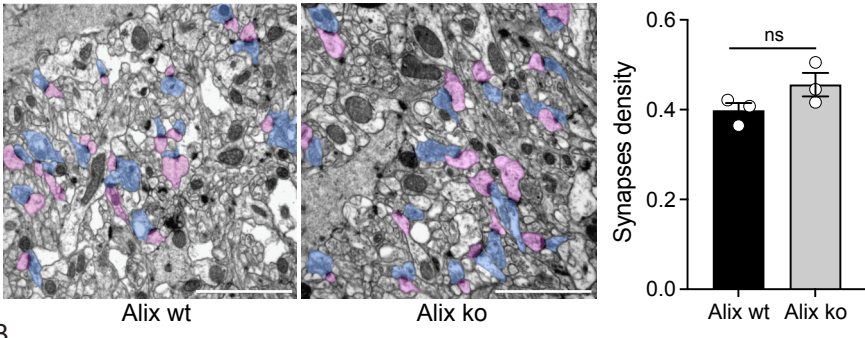

C

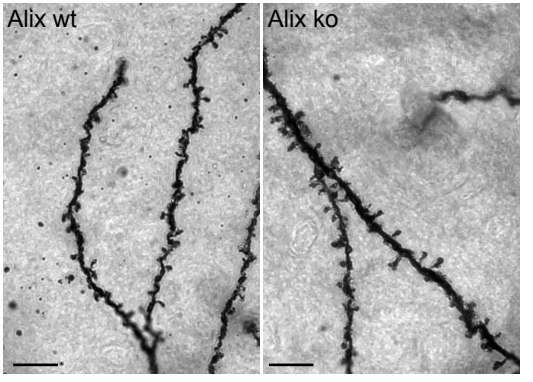

B

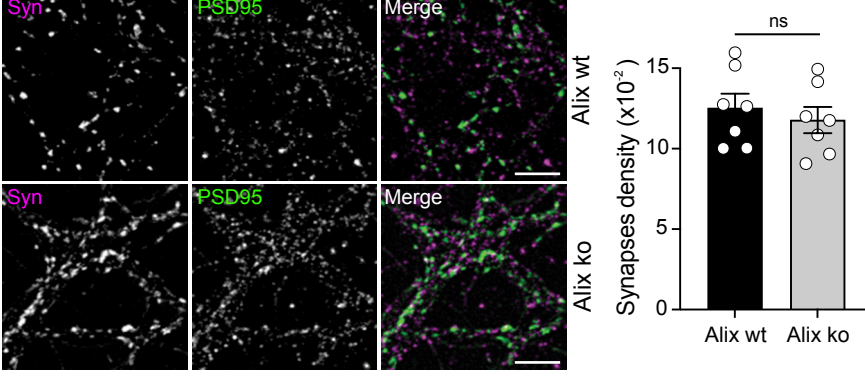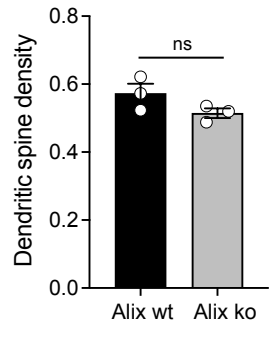

Supplement: S5 Fig — (A) Representative electron micrographs of the CA1 stratum radiatum from Alix wt and ko brain sections. Presynaptic profiles are highlighted in blue, and dendritic spines are in purple. Scale bar: 2 μm. Graph shows no difference in synaptic density per μm2 of Alix wt and ko brains. (B) 15 DIV hippocampal neurons were stained with anti-PSD95 (magenta) and anti-synapsin-1 (green) antibodies. Immunolabeled objects were considered as synapses when both stainings were juxtaposed. Scale bar: 10 μm. Graph shows no difference in the number of synapses per μm2 of Alix wt and ko neurons. (C) Brain sections from 8-week-old Alix wt or ko were stained by the Golgi–Cox impregnation technique. Stained dendritic segments were visualized by bright-field microscopy. Scale bar: 10 μm. Numbers of spines per μm of dendrites were counted. Average +/‒ SEM, N, statistical analysis: (A) 0.40 +/‒ 0.02; 0.45 +/‒ 0.03 for Alix wt and Alix ko, respectively. N = 600 synapses from 3 animals per genotype. Alix wt vs. Alix ko, p = 0.1395, unpaired t test. (B) 0.13 +/‒ 0.01; 0.12 +/‒ 0.01 for Alix wt and ko, respectively. N = 7 independent experiments. Alix wt vs. Alix ko, p = 0.5501, unpaired t test. (C) 0.57 +/‒ 0.23; 0.51 +/‒ 0.01 for Alix wt and Alix ko, respectively. N = 3 animals per genotype. Alix wt vs. Alix ko, p = 0.1414, unpaired t test. The data underlying all the graphs shown in the figure can be found in S1 Data. Alix, ALG-2-interacting protein X; DIV, day in vitro; ko, knockout; wt, wild type. (PDF) [file pbio.3001659.s005.pdf]

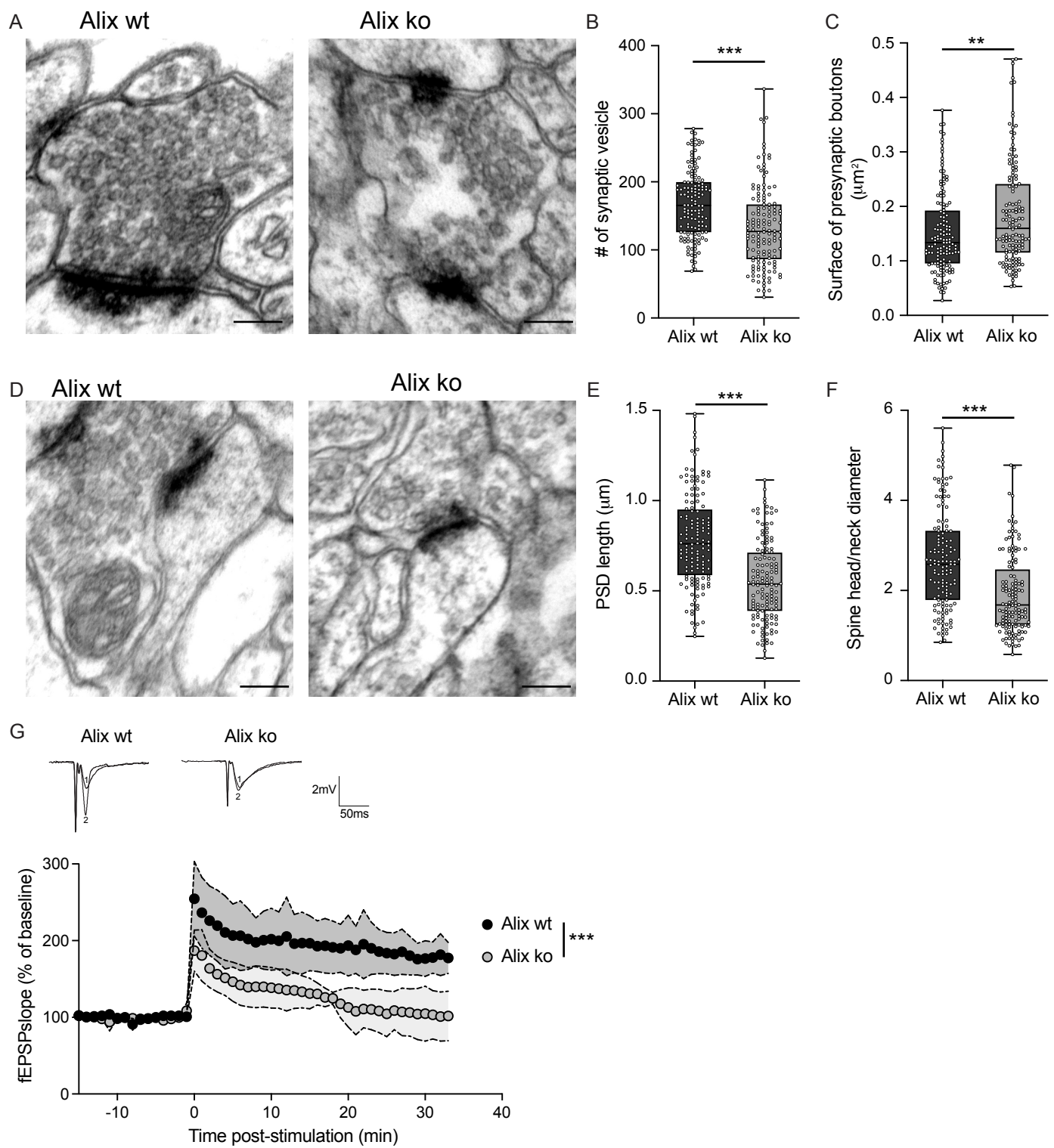

Supplement: S6 Fig — (A–D) Representative electron micrographs of CA1 from Alix wt and ko mice. Scale bar: 200 nm. (B, C, E, F) Graphs represent the numbers of SVs per μm2 (B), presynaptic bouton surface area (C), PSD length (E), ratio between the diameter of the spine head and neck (F) from sections throught the synapses. (G) LTP of fEPSP slope evoked by high frequency stimulation of Schaffer collaterals delivered at time 0. Inserts show representative EPSPs traces. Median (min to max), N, statistical analysis (B) 165.4 (69 to 278); 127.3 (31 to 336) for Alix wt and Alix ko, respectively. N = 136 synapses from 3 animals. Alix wt vs. Alix ko, p = 0.0009, Mann–Whitney test. (C) 0.13 (0.027 to 0.376); 0.16 (0.053 to 0.47) for Alix wt and Alix ko respectively. N = 136 synapses from 3 animals. Alix wt vs. Alix ko, p = 0.0036, Mann–Whitney test. (D) 0.76 (0.25 to 1.48); 0.54 (0.13 to 1.11) for Alix wt and Alix ko, respectively. N = 136 synapses from 3 animals. Alix wt vs. Alix ko, p = 0.0001, Mann–Whitney test. (E) 2.58 (0.85 to 5.60); 1.68 (0.58 to 4.78) for Alix wt and Alix ko, respectively. N = 136 synapses from 3 animals. Alix wt vs. Alix ko, p = 0.0001, Mann–Whitney test. (G) Average (10 last points): 182.9 +/‒ 6.68% and 130.7 +/‒ 2.08% for Alix wt and Alix, respectively. N = 5 slices from 3 animals per genotype, p = 0.0001, 2-way ANOVA. The data underlying all the graphs shown in the figure can be found in S1 Data. Alix, ALG-2-interacting protein X; EPSC, excitatory postsynaptic current; fEPSP, field excitatory postsynaptic potential; LTP, long-term potentiation; ko, knockout. (PDF) [file pbio.3001659.s006.pdf]

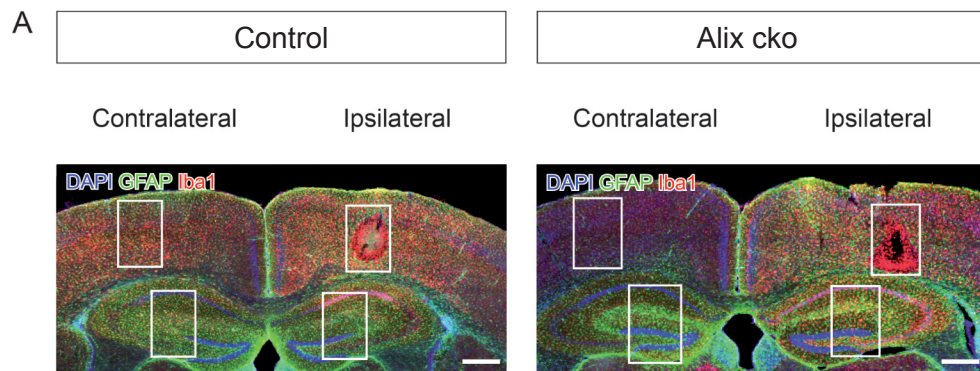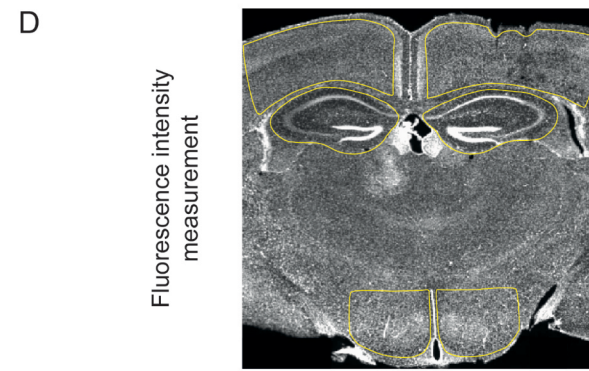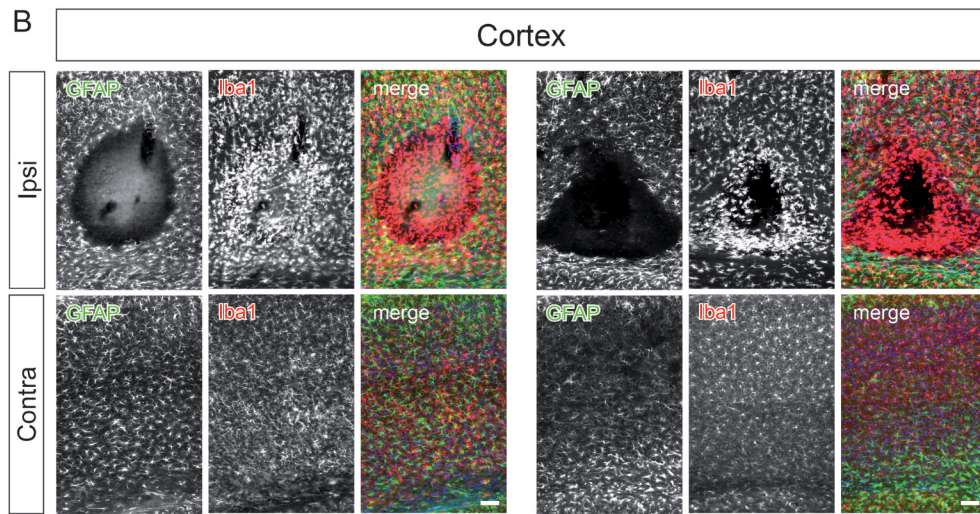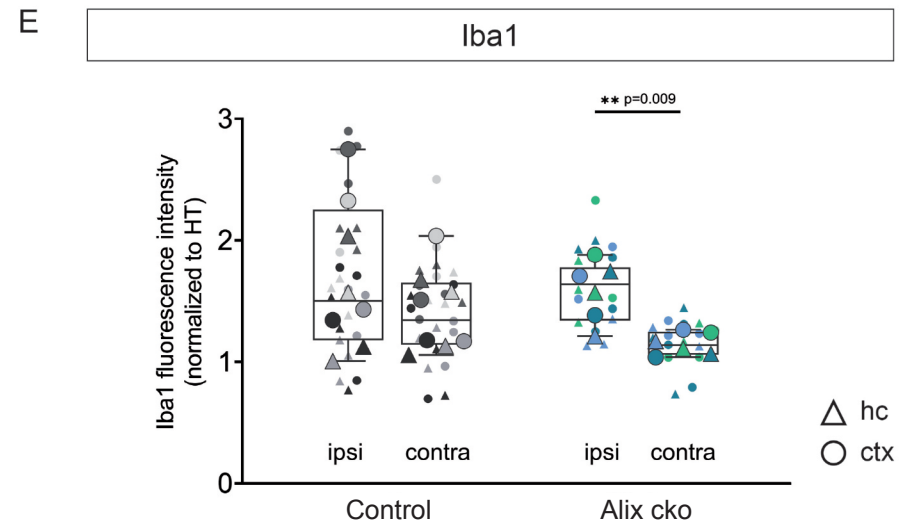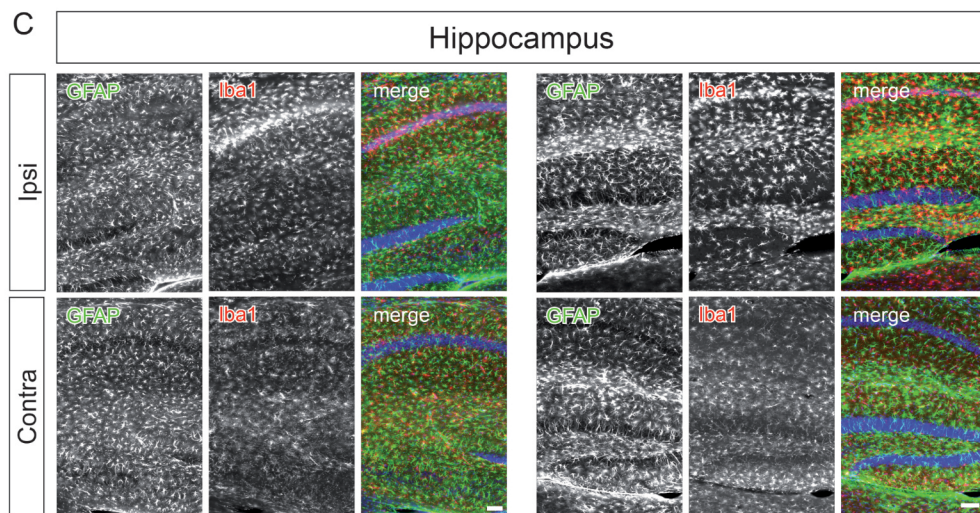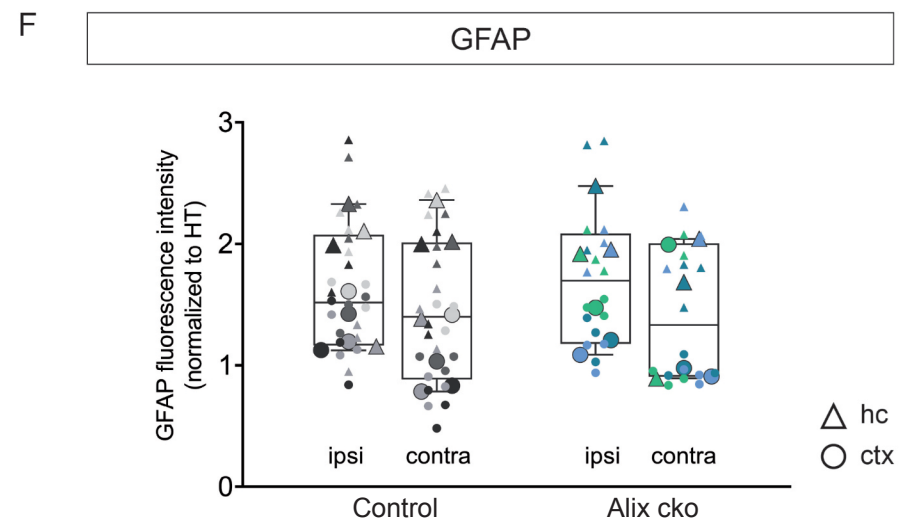

Supplement: S7 Fig — (A) Coronal brain section from control or Alix cko stained for DAPI (blue), GFAP (green), and Iba1 (red). Twenty-four-h post kainate injection. Scale bar = 500 μm. (B, C) Magnification of highlighted cortical (B) and hippocampal (C) areas show an increased ipsilateral (ipsi) microglial activation (Iba1) compared to contralateral (contra), which was more pronounced in Alix ko mice. Astroglial reactivity (GFAP) was moderately increased adjacent to the injection site in both groups. Scale bars = 100 μm. (D) Depiction of fluorescence intensity measurement areas in cortex, hippocampus, and hypothalamus. (E) In Alix ko mice, the contralateral Iba1 immunoreactivity was about 30% reduced in comparison to ipsilateral, in contrast to control mice. (F) GFAP immunoreactivities were not significantly different between hemispheres and experimental groups, as expected from the early analysis time point of only 24 h after kainate injection. Cortical GFAP immunoreactivity (A) was similar to hypothalamus (normalized fluorescence intensity ≈ 1). The hippocampal GFAP expression (B) showed a higher variability, however, approximately twice as high (normalized fluorescence intensity≈ 2) compared to cortex and hypothalamus. These data reflect the regional heterogeneity in astroglial GFAP expression, acting as internal confirmation of the analysis method. Circles and triangles represent individual quantifications from cortex and hippocampus, respectively. Large data points correspond to the average of 3 slices from the same animal (small data points in the background, color coded per animal). Fluorescence intensity values were normalized to the hypothalamic area (HT) of the respective hemisphere. Mean +/‒ IQR, N, statistical analysis: (E) Control: 1.1 (0.2) vs. 1.6 (0.4), contra- vs. ipsilateral medians (IQR); **p = 0.009; Alix cko: 1.3 (0.5) vs. 1.5 (1.1), contra- vs. ipsilateral medians (IQR); p = 0645. N (control) = 4 animals, N (Alix ko) = 3 animals. (F) control: 1.4 (1.1) vs. 1.5 (0.9 [file pbio.3001659.s007.pdf]
