# Supplementary material for: Alix is required for activity-dependent bulk endocytosis at brain synapses
Source: PLoS Biol. 2022 Jun 3;20(6):e3001659. doi: 10.1371/journal.pbio.3001659 (PMC9200306; doi:10.1371/journal.pbio.3001659)
Supplement: S1 Raw images — Uncropped, non-modified western blot films used in Fig 1A. Note that different exposures were used depending on the immunodetected proteins. Full western blot from Fig 1B. Uncropped, non-modified western blot films used in Fig 1B. Note that different exposures were used depending on the immunodetected proteins. (PPTX) [file pbio.3001659.s011.pptx]

## Slide 1
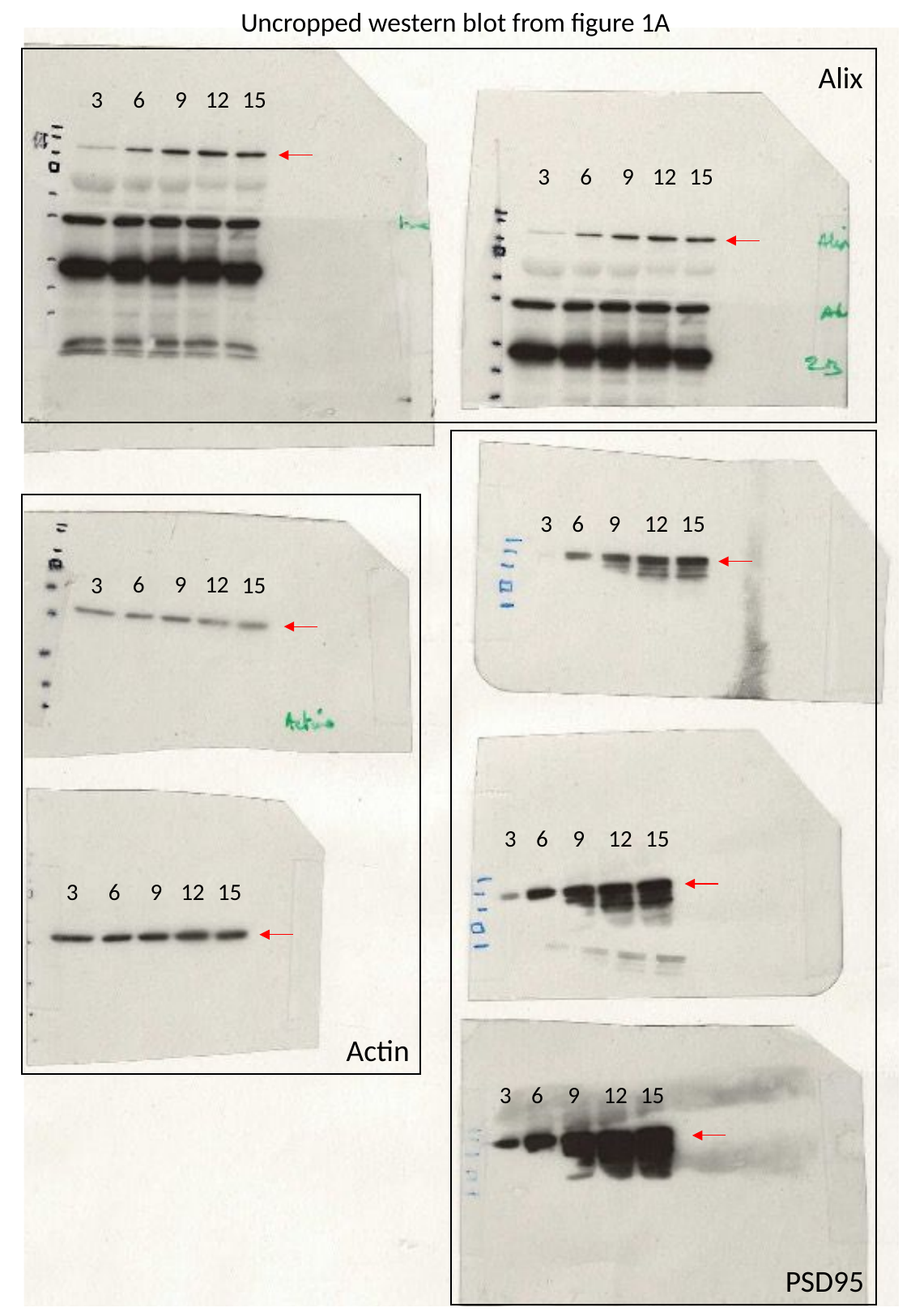

Uncropped western blot from figure 1A
Alix
6
9
12
3
15
6
9
12
3
15
6
9
12
3
15
6
9
12
3
15
6
9
12
3
15
6
9
12
3
15
Actin
6
9
12
3
15
PSD95

## Slide 2
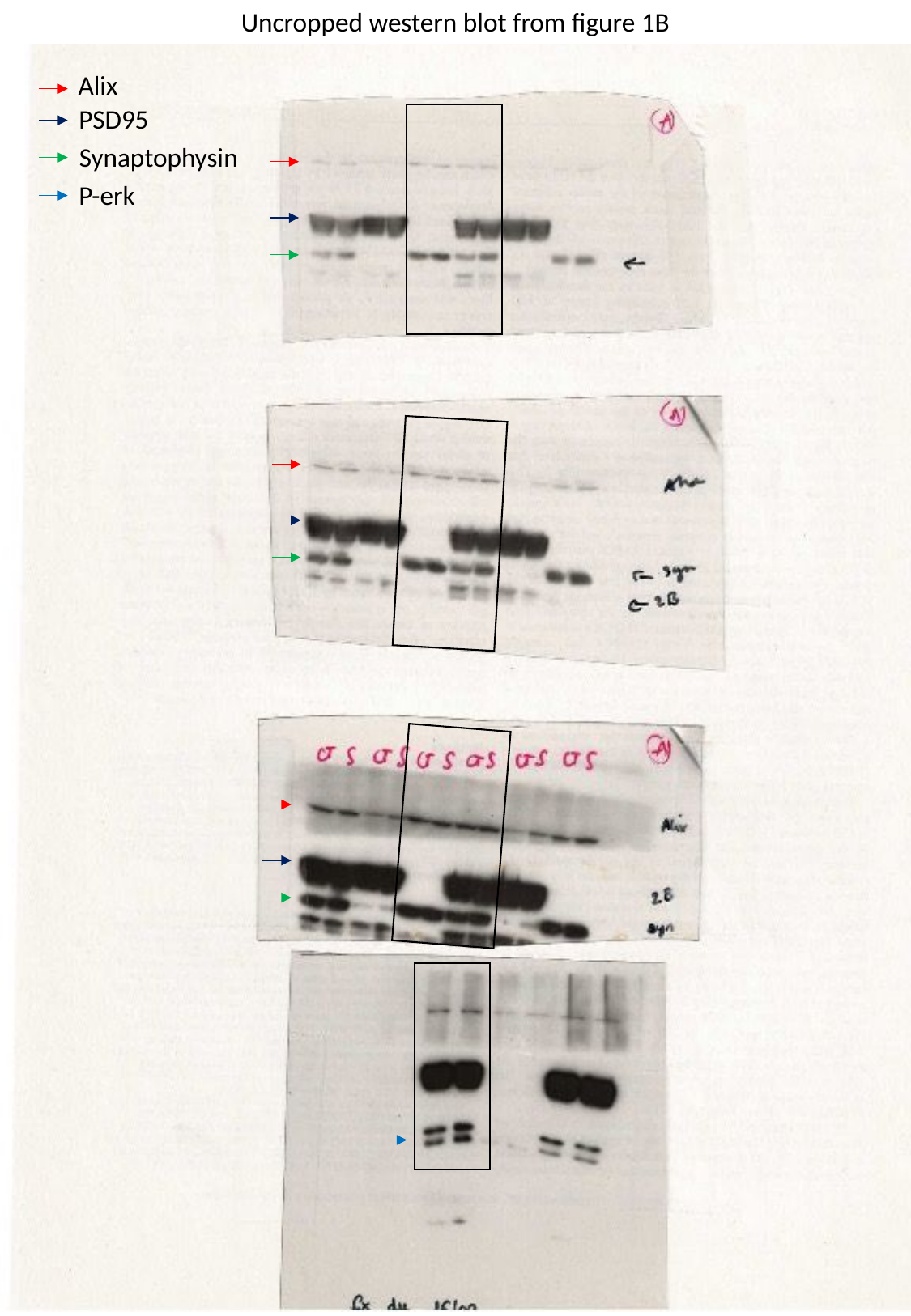

Uncropped western blot from figure 1B
Alix
PSD95
Synaptophysin
P-erk
