# Supplementary material for: Alix is required for activity-dependent bulk endocytosis at brain synapses
Source: PLoS Biol. 2022 Jun 3;20(6):e3001659. doi: 10.1371/journal.pbio.3001659 (PMC9200306; doi:10.1371/journal.pbio.3001659)
Supplement: Attachment — Submitted filename: 118987_1_rebuttal_2190904_r0bd7t.docx [file pbio.3001659.s013.docx]

Response to reviewers of Laporte et al. 27 sept 2021

Reviewer #1 (Evidence, reproducibility and clarity (Required)):

 Activity-dependent bulk endocytosis is the major endocytosis pathway in central nerve terminals during high frequency stimulation. However, the molecular mechanism remains enigmatic, limiting investigations regarding its physiological role. This paper identifies a molecule essential for this pathway and also potential downstream mechanism (however only a role for Alix is directly demonstrated here, and not ALG-2 or endophilin). The work agrees well with the current literature and provides an alternative calcium-dependent mechanism via which ADBE could be triggered. The links to epilepsy are interesting, however to be of higher significance to the disease field a more rigorous investigation of the phenotype (and the role of Alix/ADBE in inhibitory neurotransmission) would need to be performed. Regardless, the work will be of considerable interest for researchers working in synaptic communication and neurotransmitter release.

On the whole this appears to be a well-executed study with good controls and appropriate analysis. The methods used to address the research questions are appropriate and the findings appear robust. The key conclusions are convincing, with one exception which I have outlined below. This will need one additional experiment, which should be simple to perform (providing the Alix mouse line is still available). The data and methods are on the whole well presented, although I have a number of more minor comments below. The n numbers and stats appear fine. 

**Major points**

 -My only major concern is the method of stimulation used in the majority of the cell culture experiments. The authors show convincingly that that dextran uptake is abolished in hippocampal neurons using bicuculline/4AP stimulation. However they also see less bursting behaviour in Alix KO cultures, suggesting these neurons are not receiving the same stimulus as wild-type. This reduction in activity may affect the extent of ADBE, since it is an activity-dependent endocytosis mode. Therefore it could be argued that the reduction in ADBE is due to the reduced excitability of Alix KO rather than Alix having a direct role. To address this, it would be good to demonstrate a block of ADBE using a method of stimulation that evokes an equal depolarization between wild-type and KO neurons. Electrical field stimulation or even elevated KCl should hopefully provide this. I appreciate the HRP uptake studies were performed with KCl, however these were performed in cerebellar granule cells which are usually hyperpolarised (since they are cultured in depolarizing media). Therefore to confirm that differential excitability between WT and KO neurons is not the reason for the ADBE phenotype, the dextran assay (and ideally HRP uptake) should be repeated in hippocampal neurons using an alternative stimulation method.

Before answering this very important remark of Reviewer 1, we need to mention the following: R. Sadoul is now working at the Institute of Structural Biology (Grenoble) which has no neuronal cell-culture facilities or animal house. This explains why Alix KO mice are now only raised in the laboratory of F. Kirchhoff (Homburg), who performed most of the *in vivo* and hippocampal slices experiments. However, the Kirchhoff’s lab has no experience with hippocampal cell culture and the two PhD students who performed the cell culture experiments quantifying ADBE have now left the Sadoul’s lab and will therefore not be able to do the experiments in Kirchhoff’s lab. Therefore, comparing ADBE between KO and WT using a different stimulation than Bic/4AP would require at least 3 to 4 months of work, taking the risk of loosing the novelty of our finding. However, we feel that the results described in the manuscript answer the concerns of Reviewer 1 that Alix KO and WT neurons have different levels of excitability. The arguments showing that this is not the case are the following:

1) Figure 3A, B show no obvious difference between the increase in Syp-pH exocytosis at the level of individual synapses, which would be the case if there were major defects in synaptic release of Alix KO neurons. In order to reinforce this, we would like to add in the revised manuscript a quantification of the exocytosis rates under these conditions.

2) In our hippocampal neurons, ADBE revealed by dextran uptake is triggered by increasing electrical activity with bic/4AP. We took special care to showing that under these circumstances, bic/4AP increases sufficiently Alix KO neuron activity. As this reviewer pointed out, electrical activity recordings show that basic activity reflected by the number of bursts/ min is lower in KO neuronal cultures (Figure S3C). However, as seen on the graph below, bic/4AP stimulation increased the response in KO neurons by a factor which is even bigger than that in WT neurons. This additional quantification also shows that this higher response to bic/4AP lasts with time.

Additional evidence that KO neurons respond similarly to bic/4AP stimulation is provided in Figure S1A, which shows identical calcium increase upon stimulation in both WT and KO neurons. Altogether, these data indicate that even if Alix KO neurons fire less under normal conditions, they react more strongly to bic/4AP stimulation which is used to trigger ADBE. Therefore, the absence of ADBE observed in Alix KO neurons is unlikely due to a reduced effect of bic/4AP on the activity of the neuronal culture.

**Minor points**

1)Fig 1C - It would be good to see the proportion of syp-pH synapses that were responsive to stimulation that also displayed recruitment of mCherry-Alix. Showing 2 illustrative examples doesn't provide information on whether this is a common phenomenon.

Quantification of the percentage of synapsin-positive synapses in which Alix had been recruited following a 5 min bic/4AP-incubation has been done on fixed neurons expressing YFP-Alix. Using a threshold value at 4 standard deviation above the mean fluorescence intensity measured at synapses of unstimulated neurons, we found a significant recruitment at 37 % of synapses lying on Alix positive axon like process (n=527 stimulated synapses; n=350 unstimulated). This result will be added in the revised manuscript. Furthermore, other curves of mCherry-Alix recruitment onto syp-pH synapses already acquired will be combined to those shown on Fig1C.

2)Fig 1G - Similarly, quantification of the co-localisation of Alix with both synapsin and PSD-95 would be useful here, rather than an illustrative image.

Quantification of the colocalization/ juxtaposition between Alix and PSD95 will be done on the images that we have already acquired (>50 synapses per culture of 5 independent cultures) and results added to the revised manuscript.

3)Figure 2 - the data here are convincing, however the histograms are difficult to interpret, in terms of the combination of neuron genotype and the fluorescent protein investigated. This could be made clearer.

 We will represent individual graphs for each protein to improve the clarity of the figure.

4)Figure 3B - it is unclear how such a large change in the time constant of the syp-pH response could come from almost completely overlapping traces - this should be checked.

This apparent discrepancy also noted by Reviewer 2, comes from the fact that the curves which are presented in the figure are made from raw data, while the normalized data were used to calculate the time constant. Also, as suggested by reviewer 3, we revised statistical analysis of our data and noticed that the significance of the time constant was assessed by using an unpaired t-test. In fact, we should have used a non-parametric Mann-Witney as our data were not following a normal distribution. Noteworthy, is that even here the difference between KO and WT remains significant with a p value of 0.002 (instead of p = 0.0005 with the t-test). The curve based on normalized data and the revised statistical analysis shown below, will added in the revised manuscript.

Nevertheless, an alternative explanation for the smaller time constant is that single vesicle endocytosis pathways are upregulated in Alix KO terminals (seen with HRP), rather than a reduction in ADBE.

This was indeed our interpretation presented in the Results and Discussion:

- l.679 *“Thus, these electrophysiological results show that synapses of Alix KO neurons which lack ABDE have less SVs but increased rate of endocytosis, as already suggested in cultured neurons by the increase of Syp-pH endocytosis and of newly formed SVs detected in Alix KO-hippocampal and CB neurons, respectively.”*

- l.784 *“On the other hand, Syp-pH fluorescence and EM analysis revealed that activity dependent synaptic endocytosis is increased in absence of Alix.”*

5)Figure 3C-G - the effect on HRP endosome number in Alix KO terminals is convincing, however looking at the images it appears the remaining HRP endosomes in Alix knockout mice are much smaller (potentially explaining the increase in HRP vesicles). The diameter of these endosomes could also be measured, formation of smaller endosomes may give clues to mechanism. 
We thank the reviewer for his suggestion, which led us reveal that the size of HRP endosomes is indeed smaller in Alix KO synapses. We now provide additional quantification of the diameters of all HRP-positive structures in WT vs KO synapses (see below). The data confirm that the diameter of HRP labelled vesicles increases upon depolarization in WT, but not in KO synapses (a). Splitting the HRP-vesicles into two groups based on their diameter, allowed us to show that the diameter of depolarization-induced vesicles above 100 nm considered as endosomes, is significantly smaller in Alix KO synapses (b). This result will be added to the revised manuscript.

6)Hippocampal slices from Alix KO brains show reduced LTP (Supp Fig 5), which is a surprise, since it is established that LTP is established and maintained at CA1 synapses via postsynaptic mechanisms. The authors should discuss whether they think this effect is due to pre- or post-synaptic effects of the absence of Alix. The remaining electrophysiological phenotypes appear largely consistent with an endocytosis defect.

LTP was induced by presynaptic high frequency stimulation (4 trains delivered at 100 Hz with 5 min between each train). The impairment in LTP might thus reflect presynaptic defects of ADBE in Alix KO synapses. Further investigations are required to sustain this hypothesis: i) using electron microscopy should allow to show that the high frequency stimulations used here indeed triggered ADBE in WT but not in Alix KO synapses ii) other LTP experimental paradigm which do not require presynaptic high frequency stimulation should be tested to show that post-synaptic events underlying LTP are not impaired in Alix KO mice, iii) all supplementary experiments should also be done on conditional mice. These experiments require extensive work and time beyond the scope of our present study, but they will be discussed in the revised version of the manuscript.

7)The reduction in seizure frequency in Alix KO mice was very interesting, and in this context it is disappointing that inhibitory neurotransmission was not also assessed when glutamatergic synapses were being examined using electrophysiological field recordings. Nevertheless, it would be good for the authors to explore in the discussion how dysfunctional ADBE in across both excitatory and inhibitory circuits could reduce seizures in this model system. 
The Alix KO mice suffer from discrete early developmental problems (Laporte et al. 2017), which could have interfered with the interpretation of experiments dealing with epilepsy. This is why we have focused the epilepsy studies on conditional KO mice in which Alix is deleted only in neocortical and hippocampal excitatory neurons. Based on this we restricted our electrophysiological studies of these mice on glutamatergic transmission. However, this reviewer is right in stating that further work could help understanding if deleting Alix in excitatory neurons may influence inhibitory circuits and thereby modify responses to Kaïnate. We will add the following in the discussion “Based on our data, Alix KO in principal excitatory neurons reduces seizure burden. Therefore, we expect that Alix plays a promoting or at least sustaining role in epilepsy syndromes based on neuronal hyperexcitability or hyperactivity of excitatory circuits. A possible effect of Alix KO on inhibitory neurons/circuits in epilepsy is more complex to predict due to an ambivalent role of inhibition in epilepsy. Indeed, while medication enhancing GABAergic neurotransmission is known to supress seizure activity, synchronous firing of local interneuron subpopulations may be involved in generating seizures (Magloire et al., 2019).

Reviewer #1 (Significance (Required)):

 As stated above, the work will be of considerable interest for researchers working in synaptic communication and neurotransmitter release. I have flagged a few elements of the work that could limit wider significance, however this is still important work that identifies a new role for a molecule in an important presynaptic event.

Expertise - cell / molecular neuroscience, presynaptic function / dysfunction, synaptic vesicle recycling. 

**Referees cross-commenting**

It appears that we are all in good agreement that the work is well executed and important. I still feel the manuscript needs tightened up with more quantification and a demonstration that the phenotype is not due to reduced excitability in Alix KO neurons. However, I agree with the points noted by the other referees, in particular regarding the use of statistics as raised by referee 3. 


Reviewer #2 (Evidence, reproducibility and clarity (Required)):

The authors describe here results obtained mainly by studying neurons lacking the "ALG-2 interacting protein-X" (Alix). They investigate the quantity of Alix in relation to the stimulation of the cultures using bicuculline, and found that this protein responds to stimulation with a substantial increase in its levels. They then find that the molecules is present in presynapses, using fluorescence microscopy. Removing the protein results in a change in endocytosis kinetics, with endocytosis becoming faster (Fig. 3). At the same time, bulk endocytosis-like objects are removed, as observed by electron microscopy. The authors then re-visit and strengthen this phenotype by the analysis of dextran endocytosis (Fig. 4). They finalize the work by explaining the consequences of Alix KO on synapse morphology (Fig. 5), on neuronal release properties (Fig. 6) and on the tendency of the animals to suffer epileptic episodes (Fig. 7). 

I find the manuscript convincing and interesting. I only have a few minor comments: 

-In Fig. 1B, the bar+error graph should be replaced with a bar that also indicates the individual experimental values, as in Fig. 1F

We will change the graph for one showing individual dataset overlapping the dynamite plot.

-Is the example shown in Fig. 2A typical? There ALG-2 seems to be mostly postsynaptic, not presynaptic. 
The chosen image is meant to show that ALG-2 is inside the same process as synapsin, indicating that ALG-2 is inside an axonal process. To confirm this and convince this reviewer we show part of the image of Figure 2A in which the synapsin channel (magenta) was oversaturated demonstrating the presence of ALG-2 (green) in the same process as synapsin.

In figure 2A of the revised manuscript, we will outline the axon on the synapsin channel to make the observation easier.

-In Fig. 3B, the two curves (WT, KO) appear to be almost identical. Where is the difference in the quantification coming from?

The following answer was given to Reviewer 1 who had the same concern:

This apparent discrepancy comes from the fact that the curves which are presented in the figure are made from raw data, while the normalized data were used to calculate the time constant. Also, as suggested by reviewer 3, we revised statistical analysis of our data and noticed that the significance of the time constant was assessed by using an unpaired t test. In fact, we should have used a non-parametric Mann-Witney as our data were not following a normal distribution. Noteworthy, is that even here the difference between KO and WT remains significant with a p value of 0.002 (instead of p = 0.0005 with the t test). The curve based on normalized data and the revised statistical analysis (see below) will added in the revised manuscript.

-In Fig. 5, were the data obtained from 3D reconstructions, or from sampling single sections? This should be clarified in the figure legend. What does the "surface area" mean? Is this the surface of the bouton, from a 3D reconstruction, or the surface of the section through the bouton? The two measurements have very different meanings, so this should be clarified by the authors. 
Data are obtained from single sections; the surface area expressed in nm^2^ represents the surface of the bouton from one section through the bouton. This will be specified both in the figure legend and MM. We reckon that our quantifications suffer from a lack of 3D reconstruction and that therefore the conclusion regarding the number of SVs in synapses should be taken with more caution. Accordingly, we suggest to present this figure as a supplementary figure.

-Fig. 6 and Fig. 7 could be presented in the supplement, since they do not add much to the main points of this manuscript.

We will pool Fig.6 and 7 in the revised manuscript. However, we feel as the two other reviewers, that this part is “interesting” (Reviewer 1) and represents “a nice finish” (Reviewer 3); we therefore would like to keep this figure as part of the main figures.

Reviewer #2 (Significance (Required)):

The manuscript presents a convincing view of how bulk endocytosis takes place. This is a very important aspect, which has never been convincingly addressed in the synapse endocytosis literature. I therefore see this work as novel and timely - overall excellent. 

**Referees cross-commenting**

This sounds good, I agree.

Reviewer #3 (Evidence, reproducibility and clarity (Required)):

The study by LaPorte and colleagues examine lof phenotypes associated with Alix (ALG-2 interacting protein X)/ PDCD6IP) during activity-dependent bulk endocytosis (ADBE), following seizure induction, and hippocampal-dependent synaptic function. The researchers approach the problem using cell-culture, ex vivo brain slice preparation, as well as telemetric EEG measurements in vivo. The is a wide array of techniques in the paper with important experiments using overlapping and confirmatory approaches to draw conclusions about the role of Alix function during clathrin-independent endocytic processes. The cell culture experiments are in particular quite convincing, the strategic and well thought out Alix mutants, each addressing a specific aspect of Alix biology make a strong case for Alix acting in a Ca++ dependent manner through endophilin to support bulk endocytosis. The EEG data are a nice finish to the detailed cellular imaging studies that demonstrate the effects of Alix KO on pathological processes i.e., seizures, that would be critically dependent on clathrin-independent SV recovery, the only data that seems a bit weak is the EM data in Figure 5. This reviewer was cautiously hesitant to accept the statistical findings for some of the points made. A more thorough reporting of the statistical findings would lend support to the stats test that was chosen in this figure (and a few other places). Nevertheless, it is a strong study using a battery of excellent complementary techniques. The Figures are easy to follow and make a compelling argument for the author's conclusions. This reviewer thinks this is a notable extension of their previous fibroblast and rat primary culture results and will be of interest to a broad readership. 

**Major comments**

One major critique is the fast and loose nature of statistical reporting. This reviewer would like the statistics to be reported more robustly, including normality measurements (i.e., to justify Man-Whitney over t-Tests in some figures like Figure 5). Effect size, F, and t-values should be added. 
We apologize for not explaining well enough the statistical analysis. Here are the details of the statistical analysis used in Figure 5:

Effect sizes / Cohen's D are calculated as the difference of means divided by the pooled weighted standard deviation:

$(\left| m1-m2 \right|)/\surd{((SD1}^{2}\times\frac{n1}{\left( n1+n2 \right)})+{(SD2}^{2}\times\frac{n2}{\left( n1+n2 \right)}))$.

Please note that all along the manuscript we had followed the procedures described in our Material and Methods: *“The comparison of two groups was performed using two-sided Student’s t-test or its non-parametric correspondent, the Mann-Whitney test, if normality was rejected (Shapiro-Wilks test). The comparisons of more than two groups were made using one or two way ANOVAs followed by post-hoc tests (Holm Sidak’s or Tukey’s HSD) to identify all the significant group differences. N indicates independent biological replicates. The graphs with error bars indicate 1 SEM (+/-) except for figure 5 where we used box plots showing distribution of medians (whiskers = min and max values)…”*

We will provide in the revised manuscript a table detailing figure by figure all statistical analysis including how normality was tested as well as means, confidence intervals, effect sizes, F and t-values when applicable.

**Minor comments**

Can the authors examine the EEG data with respect to sex?

 Please find a figure which allow to discriminate between males (circles) and females (triangles). Since there is no apparent difference between sexes, we wish to keep the figure as it was in the submitted manuscript.

Remove the comma "with, and" line 43

Capitalize "figures" line 597

Thanks for carefully reading, we will make these changes in the revised manuscript.

Figure 1, the pERK staining is odd, typically a pair of bands at 42 and 44 are visible, no total ERK is shown to compare phospho/total. The quantification for western shown on 1B does not match the images. A more representative image should be chosen.

We will add the images corresponding to the total Erk and update the figure in the revised manuscript following reviewer’s comment.

Supplementary Figure 1, panel B: The images do not look representative for the quantification in panel C.

We will find a more representative image to illustrate our quantification

Supplementary figure 3C: There is a typo on the graph. It should be "bursts" not "birsts"

Thanks for careful reading, we will correct the legend in the revised manuscript.

Kainate is used to induce seizures for this study. However, there are mouse lines that for example, have audiogenic seizures. These lines are used to study epilepsy. It would be intriguing to cross breed an epileptic mouse with the Alix KO mouse and directly study the effect of Alix KO on epileptic seizures. It would be interesting to know if Alix is required for different forms of seizure activity.

The following paragraph will be added in the revised discussion:
“Since epileptic network activity solicits sustained neuronal firing activity, we would predict a role for Alix-driven ADBE across epilepsy types. The degree and nature of impact, however, might vary according to the etiology of the epileptic syndrome in question. In addition to our pharmacological kainate model, it would be intriguing to analyze Alix-dependent seizure susceptibility also in genetic models with spontaneous seizures, such as mutations of the voltage-gated sodium channel SCN8A (Lozovaya et al., 2014) or tuberous sclerosis complex 1/2 (Meisler et al., 2019), as well as inbred mouse strains with high susceptibility to audiogenic seizures (DBA/1J, DBA/2J or 129/SvTer strain) (Martin et al., 2020). However, each of these lines would require its own in depth-analysis far beyond the current work.”

Reviewer #3 (Significance (Required)):

 The work extends previous findings from this author's group in they used Alix deficient cells, to determine that Alix was required for clathrin independent endocytosis (CIE) during ligand-induced endophilin-dependent endocytosis and bulk endocytosis. This study now shows the important function of Alix at synapses during rapid exocytosis and endocytosis processes using pharmacological and electrophysiological induction of synaptic release. It is a logical and reasonable movement forward from their previous observations based on observations of high concentrations of Alix in rat neurons.

**Referees cross-commenting**

Excitability is a great point, I do suggest this be addressed as an essential control. 


This work will probably be of broad appeal to researchers studying synaptic vesicle release, recycling, and synaptic function in general. It has relevance to human neurological disorders in which SV release is pathologically impacted like epilepsy and Alzheimer's disease. 

I have published and worked in this general area for 20+ years.
